# Photoplethysmographic determination of the respiratory rate in acutely ill patients: validation of a new algorithm and implementation into a biomedical device

**DOI:** 10.1186/s13613-019-0485-z

**Published:** 2019-01-21

**Authors:** Erwan L’Her, Quang-Thang N’Guyen, Victoire Pateau, Laetitia Bodenes, François Lellouche

**Affiliations:** 1 Réanimation Médicale, LATIM INSERM UMR 1101, CHRU de la Cavale Blanche, Bvd Tanguy-Prigent, 22 rue Camille Desmoulins, 29609 Brest Cedex, France; 2 Médecine Intensive et Réanimation, CHRU de la Cavale Blanche, Bvd Tanguy-Prigent, 29609 Brest Cedex, France; 3 Oxynov Inc, Technopole Brest Iroise, 135 rue Claude Chappe, 29280 Plouzané, France; 4 Centre de Recherche de l’Institut Universitaire de Cardiologie et de Pneumologie de Québec, 2725 Ch Ste-Foy, Quebec, QC G1V 4G5 Canada

**Keywords:** Respiration rate, Respiratory failure, Pulse oximetry

## Abstract

**Background:**

Respiratory rate is among the first vital signs to change in deteriorating patients. The aims of this study were to evaluate the accuracy of respiratory rate measurements using a specifically dedicated reflection-mode photoplethysmographic signal analysis in a pathological condition (PPG-RR) and to validate its implementation within medical devices.

**Methods:**

This study is derived from a data mining project, including all consecutive patients admitted to our ICU (ReaSTOC study, ClinicalTrials.gov identifier: NCT02893462). During the evaluation phase of the algorithm, PPG-RR calculations were retrospectively performed on PPG waveforms extracted from the data warehouse and compared with RR reference values. During the prospective phase, PPG-RR calculations were automatically and continuously performed using a dedicated device (FreeO_2_, Oxynov, Québec, QC, Canada). In all phases, reference RR was measured continuously using electrical thoracic impedance and chronometric evaluation (Manual-RR) over a 30-s period.

**Results:**

In total, 201 ICU patients’ recordings (SAPS II 51.7 ± 34.6) were analysed during the retrospective evaluation phase, most of them being admitted for a respiratory failure and requiring invasive mechanical ventilation. PPG-RR determination was available in 95.5% cases, similar to reference (22 ± 4 vs. 22 ± 5 c/min, respectively; *p* = 1), and well correlated with reference values (*R* = 0.952; *p* < 0.0001), with a low bias (0.1 b/min) and deviation (± 3.5 b/min). Prospective estimation of the PPG-RR on 30 ICU patients’ recordings was well correlated with the reference method (Manual-RR; *r* = 0.78; *p* < 0.001). Comparison of the methods depicted a low bias (0.5 b/min) and acceptable deviation (< ± 5.5 b/min).

**Conclusion:**

According to our results, PPG-RR is an interesting approach for ventilation monitoring, as this technique would make simultaneous monitoring of respiratory rate and arterial oxygen saturation possible, thus minimizing the number of sensors attached to the patient.

*Trial registry number* ClinicalTrials.gov identifier NCT02893462

## Introduction

Respiratory rate (RR) is among the first vital signs to change in deteriorating patients, and it has been reported to be the best individual predictor of cardiac arrest in general wards [1]. Current approaches to respiratory rate monitoring may limit their ability to detect changes in patients’ condition [2–4]. Intermittent RR measurement via visual observation is routine in many patient care settings, and it is usually estimated by visual counts of the chest wall movements for 15 s to 1 min. This approach has several inherent limitations that diminish the clinical utility of these measurements mainly because it is intermittent, and some patients’ “morphology” (e.g. obesity, thoracic deformation) may alter the ability of caregivers to detect chest or abdomen ventilatory-induced movements. Most of all, such non-continuous observation may lead to false appreciation/underestimation of clinical deterioration or clinical worsening.

The photoplethysmographic (PPG) signal is obtained by measuring the intensity of light penetrating through or reflected by the skin, and its widespread application is the routine non-invasive monitoring of arterial oxygen saturation by pulse oximetry, developed in the early 1980s [5, 6]. Recent evidences suggest that RR measurements extracted from PPG may be of clinical interest. The PPG signal includes ventilatory component [7–12] seen as modulation of the frequency [frequency modulation (FM)] and the amplitude [amplitude modulation (AM)] of the cardiac pulse but also modulation of the intensity of the PPG signal baseline [baseline modulation (BM)] (see “Appendix”). FM is caused by respiratory sinus arrhythmia (RSA), which is a cardiac vagal reflex responsible for a heart rate increase during inspiration and decrease during exhalation [11, 12]. AM is caused by cardiac stroke volume variations resulting from ventilatory influence on venous return, which decreases during exhalation and increases during inspiration in spontaneous breathing (conversely in positive ventilation) [11, 12]. Such ventilatory modulation of the PPG signal is the direct reflection of the respiratory rate [8, 9].

Several studies have attempted to extract RR from PPG recordings [11, 13–20]. However, the majority of these studies have been carried out in controlled settings using PPG recordings from small groups of healthy subjects, have included PPG waveforms without artefacts (which tends to be frequent in clinical routine), have not depicted results extracted from either a derivation and a prospective validation phase, and were performed at rest and/or in the absence of activity or critical condition such as atrial fibrillation [11, 14–17, 19, 20]. Most of all, the vast majority of these algorithms have not been implemented in any medical devices for a routine clinical validation, except one [21].

Several other technological approaches for non-invasive RR monitoring techniques have been proposed to address the limitations associated with RR-manual count. These techniques may involve end-tidal CO_2_ monitoring, nasal thermistances, respiratory inductance plethysmography via chest bands and ECG-based electrical impedance. However, beside several conflictory results of various RR extractions between these methods, as compared to clinical Ref. [22–25], the fact that some of these techniques may be considered as intrusive given the dedicated interfaces that are required, the most striking problem for a wide acceptance should be the non-ubiquity of these methods, as compared to PPG that limit their practical application in many clinical settings. While PPG is nowadays ubiquitous in most general wards or step-down facilities, its use to measure RR could facilitate early warning in case of respiratory failure occurrence, thus enabling to monitor concomitantly its two main determinants, i.e. SpO_2_ and RR.

The aims of this study were to evaluate the accuracy of respiratory rate measurements using a specifically dedicated reflection-mode PPG signal analysis in a pathological condition and to validate its implementation within medical devices.

## Methods

This study is derived from a data warehousing and data mining project, including all consecutive patients admitted to our ICU. Our local ethics committee (*Comité d’Ethique du CHRU de Brest*) approved the data collection study and waived written informed consent according to French legislation. Information was provided to all patients and relatives at ICU admission (ReaSTOC study, ClinicalTrials.gov identifier: NCT02893462).

### Patients and measurement set-up

#### Retrospective evaluation phase

During this evaluation phase of the algorithm, PPG-RR calculations were retrospectively performed on PPG waveforms extracted from the ReaSTOC physiological signals’ data warehouse and compared with RR reference values extracted from metadata files. All recordings from the ReaSTOC database were prospectively performed by the same research assistant, using a standardized protocol.

#### Prospective evaluation phase

Considering experience from the retrospective evaluation phase, the algorithm was refined and implemented in a biomedical device CE-marked (FreeO_2_, Oxynov, Québec, QC, Canada) that enables continuous parameters recordings. PPG-RR estimation with the FreeO_2_ system was performed using the standard SpO_2_ sensor of the device. For the sake of standardization, we only included spontaneously breathing patients and excluded those with unavailable PPG curves (e.g. patients with severe with hemodynamic instability and vasoconstriction).

### Measurements

#### General data recording

During all phases, measurements were taken with the patients lying in the supine position, with a 30° angulation of the bed head.

Clinical metadata (physiological parameters, clinical status, underlying pathology, medications, etc.) were recorded by the research assistants using our standard monitoring devices and the computerized medical file (ICCA^®^ Philips Healthcare, Amsterdam, the Netherlands).

In all phases, reference RR was measured continuously using both electrical thoracic impedance (ECG-RR) and chronometric evaluation (Manual-RR) over a 30-s period, under spontaneous ventilation or ventilatory rate (Ventilator-RR) for patients under ventilatory assistance. We considered as a reference the mean RR value over three consecutive measurements, taken each 15 min, during a mean 60-min duration, by a dedicated research assistant.

#### Features extraction

##### Retrospective evaluation phase

During the retrospective phase, we used a dedicated network that enable direct recording of raw physiological waveforms (ECG at 500 Hz, PPG at 125 Hz and pressure at 62.5 Hz) and a specific data extraction software (SYNAPSE system version 1, LTSI—INSERM U1099, Rennes, France).

After a resting period of 5–10 min, measurements were taken each 15 min during a mean 60-min recording period, for each patient newly admitted to our ICU. Participants were breathing either spontaneously or under mechanical ventilation.

At least three 2-min PPG periods, free from movement-induced disturbances, were selected from each measurement.

The PPG signal includes respiratory synchronous components, seen as frequency modulation of the heart rate (respiratory sinus arrhythmia), amplitude modulation of the cardiac pulse and respiration-induced intensity variation in the PPG baseline. PPG-RR value is extracted via the analysis of respiration-induced frequency components embedded in standard PPG waveform. Advanced statistical signal processing techniques were used to enhance these components and thus reduce the effect of unavoidable disturbance such as artefacts or transient and spontaneous phenomena. A mean PPG-RR value was provided and compared to the mean reference value over the 60-min period.

##### Prospective evaluation phase

During the prospective evaluation phase, PPG-RR calculations were performed using PPG signals extracted from the Nonin PureSAT^®^ internal OEM III (Plymouth, MN, USA) of the FreeO_2_ system (Oxynov Inc., Québec, QC, Canada), using a standard reusable Nonin PureLight^®^ finger clip sensor, positioned on the index, middle or ring finger. The algorithm that was used was derived from the previous analysis phase.

PPG-RR calculations were performed at the patients’ bedside during a 45-min period of PPG signal stability, using the dedicated device. Continuous calculations were performed each respiratory cycle, within a 10-min moving average window. PPG-RR was considered as the mean value during the overall 60-min period. Manual-RR count was performed concomitantly to the PPG signals recording.

### Statistical analysis

Chronometric measurements of the respiratory rate (Manual-RR), electrical thoracic impedance (ECG-RR) and ventilatory respiratory rate values (Ventilator-RR) were regarded as the reference methods for respiration rate assessment and plethysmographic calculations (PPG-RR) as the method of comparison. Linear correlation between the different methods was performed using Pearson’s correlation coefficient analysis; Intraclass correlation coefficient (ICC) for average measurements is presented as a single-number summary that was used to assess reliability of different measurements averaged together [26].

The agreement between quantitative measure of the reference and the estimated RR values (PPG-RR) was then graphically appreciated according to Bland and Altman [27].

Statistical significance for a bilateral test was set a priori at *p* < 0.01 for all relevant analyses. Analyses were performed using MedCalc Statistical Software version 18.6 (MedCalc Software bvba, Ostend, Belgium).

## Results

### Retrospective evaluation phase

A total of 201 ICU patients’ recordings were analysed during the retrospective evaluation phase. Patients’ physiological characteristics are given in Table 1.

**Table 1:** Physiological characteristics of the patients—retrospective phase

General characteristics (*n* = 201)	
Age (mean ± SD)	61.7 ± 14.5
Sex ratio (F/M)	62/137
BMI (kg/m^2^; mean ± SD)	27 ± 6
SAPS II (mean ± SD)	51.7 ± 18.8
Chronic respiratory disorder *n*-(%)	49 (24.3)
Immunodepression *n*-(%)	14 (7.1)
Admission diagnosis	
Respiratory failure *n*-(%)	83 (41.1)
Cardiovascular failure *n*-(%)	56 (27.7)
Neurological failure *n*-(%)	36 (17.8)
Other *n*-(%)	27 (13.4)
ICU length of stay (day; mean ± SD)	12.8 ± 14.1
Hospital length of stay (day; mean ± SD)	28.7 ± 31.4
Overall survival rate *n*-(%)	135 (67.8)
Respiratory conditions	
Respiratory rate (c/min; mean ± SD)	22 ± 6
SpO_2_ (%; mean ± SD)	95 ± 4
PaO_2_/F_I_O_2_ or SpO_2_/F_I_O_2_ (mean ± SD)	269 ± 126
Invasive MV *n*-(%)	128 (63.7)
IV sedation *n*-(%)	87 (43.3)
Paralysing agents *n*-(%)	31 (15.4)
Tidal volume (mL/kg IBW; mean ± SD)	7.1 ± 2.0
PEEP (cm H_2_O; mean ± SD)	5.3 ± 2.6
F_I_O_2_ (%; mean ± SD)	38 ± 19
Patient/ventilator asynchrony *n*-(%)	28 (21.9)
Spontaneous breathing *n*-(%)	73 (36.3)
Standard oxygen	66 (32.8)
Non-invasive ventilation	7 (3.5)
HFNO *n*-(%)	5 (2.5)
Hemodynamic conditions	
Heart rate (b/min; mean ± SD)	95 ± 22
Mean arterial blood pressure (mmHg; mean ± SD)	83 ± 16
Lactate (mmol/L; mean ± SD)	2.8 ± 3.4
Atrial fibrillation	32 (16.8)
Vasopressive agent	57 (28.4)
Hemodynamic instability	29 (14.4)

Values are provided as *n*-(%) or mean ± SD, unless specified otherwise

Respiratory rate is provided as the reference method (chronometric measurement measured over 30 s under spontaneous ventilation or ventilatory rate for patients under ventilatory assistance)

Chronic respiratory disorders are considered for a previous medical history of COPD, OSA and chronic respiratory insufficiency

Oxygenation parameters are provided as PaO_2_/F_I_O_2_ for patients under mechanical ventilation or as SpO_2_/F_I_O_2_ for patients under spontaneous ventilation

Hemodynamic instability was defined as a need for fluid challenge (≥ 500 mL) and/or vasopressive agent increase during the past 2 h of care

BMI, body mass index; SAPS II, Simplified Acute Physiology Score version 2; ICU, intensive care unit; SpO_2_, pulse oximetry saturation; PaO_2_, partial pressure of oxygen in arterial blood; FIO_2_, inspiratory fraction of oxygen; IV, intravenous; PEEP, positive end-expiratory pressure; HFNO, high-flow nasal oxygen

Patients’ condition was severe (mean SAPS II 51.7 ± 34.6), and most of them were admitted for a respiratory failure and required invasive mechanical ventilation.

Hemodynamic instability and atrial fibrillation were present for a significant amount of patients (14.1% and 16.1%, respectively), and vasopressive agents were used in almost 30% patients.

**Fig. 1:**
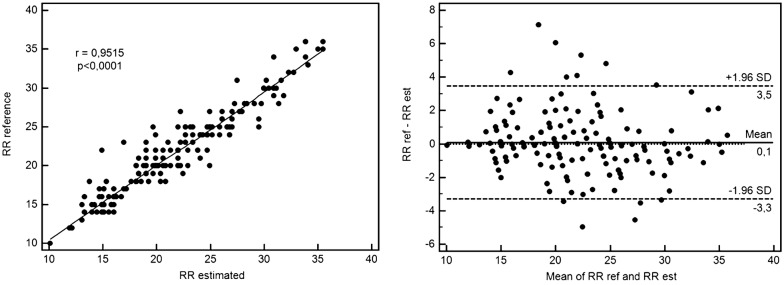
Correlation and Bland–Altman plot for reference and estimated respiratory rate during the retrospective evaluation phase. Reference respiratory rate (RR) values are provided as the chronometric measurement measured over 30 s under spontaneous ventilation or as the ventilatory rate for patients under ventilatory assistance. Estimated RR-PPG was performed using the plethysmographic curve analysis. On the 201 ICU patients’ recordings, retrospective estimation of the RR-PPG was highly correlated with the reference method (*r* = 0.95; *p* < 0.0001). Comparison of the methods using the Bland–Altman method depicted a low bias (0.1 c/min) and deviation (< ± 3.5 c/min)

PPG-RR determination was available in 95.5% cases (Table 2). Mean PPG-RR estimated value was similar to reference (22 ± 4 vs. 22 ± 5 c/min, respectively; *p* = 1), well correlated with reference values (*R* = 0.952; *p* < 0.0001), with a low bias (0.1 c/min) and deviation (± 3.5 c/min) (Fig. 1). The correlation was similar for mechanically ventilated or spontaneously breathing patients (*R* = 0.952 and 0.940, respectively; *p* < 0.0001), and those with or without atrial fibrillation (*R* = 0.916 and 0.954, respectively; *p* < 0.0001), but estimation errors were predominant in patients with atrial fibrillation, with a lower intraclass correlation coefficient.

**Table 2:** Respiratory measurements according to different techniques—retrospective phase

Number of patients with reference values	
Manual-RR *n*-(%)	199/201 (99.0)
Ventilator-RR *n*-(%)	128/128 (100)
PPG-RR *n*-(%)	193/201 (96.0)

Measurements (c/min)				
Reference RR	Estimated RR-PPG	*R*	ICC	*p*
22 ± 4	22 ± 5	0.955	0.931	< 0.0001

Reference RR under MV	Estimated RR-PPG under MV			
21.9 ± 5.4	21.8 ± 5.6	0.952	0.793	< 0.0001
With VT > median	With VT > median			
20.1 ± 3.9	20.0 ± 4.1	0.947	0.969	< 0.0001
With VT ≤ median	With VT ≤ median			
22.6 ± 6.1	22.5 ± 6.2	0.974	0.886	< 0.0001

Reference RR under SB	Estimated RR-PPG under SB			
23.2 ± 5.8	22.9 ± 5.9	0.940	0.963	< 0.0001

Reference RR without AF	Estimated RR-PPG without AF			
22.1 ± 5.6	22.0 ± 5.8	0.954	0.973	< 0.0001

Reference RR with AF	Estimated RR-PPG with AF			
20.6 ± 3.7	20.5 ± 4.0	0.916	0.689	< 0.0001

Values are provided as *n*-(%) or mean ± SD (b/mn), unless specified otherwise. R, Pearson’s correlation coefficient; ICC, intraclass correlation coefficient for average measurements, was assessed to evaluate the reliability of measurements; a *p* value < 0.01 was considered statistically significant for a bilateral test

Reference RR values are provided as the chronometric measurement measured over 30 s under spontaneous ventilation or as the ventilatory rate for patients under ventilatory assistance

RR, respiratory rate; PPG, plethysmographic measurement; Manual-RR, chronometric evaluation of the RR, over a 30-s period; Ventilator-RR, RR value provided by the ventilator in case of mechanical ventilation; PPG-RR, estimated respiratory rate using the plethysmographic curve analysis; MV, mechanical ventilation; SN, spontaneous breathing; AF, atrial fibrillation; VT, tidal volume (median tidal volume = 475 mL)

### Prospective evaluation phase during spontaneous ventilation

**Fig. 2:**
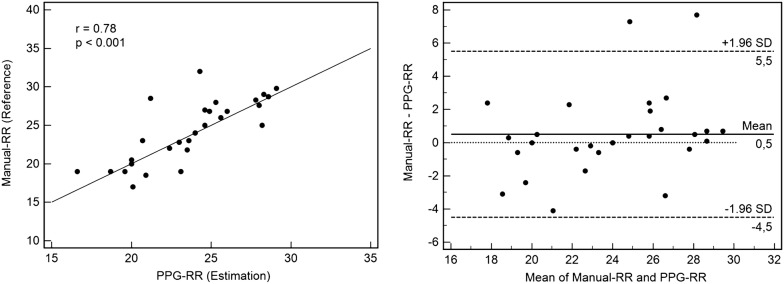
Correlation and Bland–Altman plot for reference and estimated respiratory rate during the prospective evaluation phase. Reference respiratory rate (RR) values are provided as the chronometric measurement measured over 30 s under spontaneous ventilation or as the ventilatory rate for patients under ventilatory assistance. Estimated RR was performed using the plethysmographic curve analysis (RR-PPG). On the 30 ICU patients’ recordings, prospective estimation of the RR-PPG was well correlated with the reference method (*r* = 0.78; *p* < 0.001). Comparison of the methods using the Bland–Altman method depicted a low bias (0.5 c/min) and acceptable deviation (< ± 5.5 c/min). Most of the estimation errors were related to difficult PPG signal acquisition, mostly due to artefacts and/or bad signals

Thirty patients were recorded and analysed during this prospective evaluation phase. Patients’ physiological conditions are quite similar to the retrospective phase, but patients tended to be less severe and to require less frequently respiratory assistance (Table 3). PPG-RR estimation was available in all cases, while ECG-RR was only available in 93% cases. On the 30 ICU patients’ recordings, prospective estimation of the PPG-RR was well correlated with the reference method (Manual-RR; *r* = 0.78; *p* < 0.001). Comparison of the methods depicted a low bias (0.5 c/min) and acceptable deviation (< ± 5.5 c/min). Most of the estimation errors were related to difficult PPG signal acquisition, mostly due to artefacts and/or bad signals (Fig. 2).

**Table 3:** Physiological characteristics of the patients—prospective phase (*N* = 30 patients)

Physiological characteristics	
Age (mean ± SD)	61.4 ± 11.8
Sex ratio (F/M)	11/19
BMI (kg/m^2^; mean ± SD)	27.1 ± 7.0
SAPS II (mean ± SD)	41.8 ± 16.8
Chronic respiratory disorder *n*-(%)	10 (33.3)
Immunodepression *n*-(%)	2 (6.7)
Admission diagnosis	
Respiratory failure *n*-(%)	23 (76.7)
Cardiovascular failure *n*-(%)	1 (3.3)
Neurological failure *n*-(%)	3 (10.0)
Other *n*-(%)	2 (6.6)
ICU length of stay (day; mean ± SD)	16.3 ± 15.5
Hospital length of stay (day; mean ± SD)	27.9 ± 20.8
Overall survival rate *n*-(%)	22 (73.3)
Respiratory conditions	
Mechanical ventilation *n*-(%)	10 (33.3)
Spontaneous ventilation *n*-(%)	20 (66.6)
SpO_2_ (%; mean ± SD)	93.1 ± 2.5
PaO_2_/F_I_O_2_ or SpO_2_/F_I_O_2_ (mean ± SD)	311 ± 79
Sedation *n*-(%)	6 (20)
Hemodynamic conditions	
Heart rate (b/min; mean ± SD)	94.2 ± 18.9
Mean arterial blood pressure (mmHg; mean ± SD)	87.2 ± 19.2
Atrial fibrillation	0 (0)
Vasopressive agents *n*-(%)	5 (16.7)
Hemodynamic instability *n*-(%)	2 (6.7)
Number of patients with reference values	
RR-Manual (reference) *n*-(%)	30/30 (100)
RR-ECG *n*-(%)	28/30 (93.3)
RR-PPG *n*-(%)	30/30 (100)
Measurements	
Reference RR (c/min)	22 ± 4
Estimated RR-PPG (c/min)	22 ± 5
Estimated RR-ECG (c/min)	23 ± 6

Values are provided as *n*-(%) or mean ± SD, unless specified otherwise

Reference RR values are provided as the chronometric measurement measured over 30 s under spontaneous ventilation or as the ventilatory rate for patients under ventilatory assistance

Chronic respiratory disorders are considered for a previous medical history of COPD, OSA and chronic respiratory insufficiency

Oxygenation parameters are provided as PaO_2_/F_I_O_2_ for patients under mechanical ventilation or as SpO_2_/F_I_O_2_ for patients under spontaneous ventilation

Hemodynamic instability was defined as a need for fluid challenge (≥ 500 mL) and/or vasopressive agent increase during the past 2 h of care

BMI, body mass index; SAPS II, Simplified Acute Physiology Score version 2; ICU, intensive care unit; SpO_2_, pulse oximetry saturation; PaO_2_, partial pressure of oxygen in arterial blood; FIO_2_, inspiratory fraction of oxygen; IV, intravenous; PEEP, positive end-expiratory pressure; RR-Manual, chronometric measurement of the respiratory rate; RR-PPG, estimated respiratory rate using the plethysmographic curve analysis; RR-ECG, evaluation of the respiratory rate on the electrocardiogram, using electrical impedance

## Discussion

Our work presents a feasible option to provide continuous RR monitoring in various types of patients and clinical settings. The observed performance of this technology suggests that it may be a reliable adjunct to pulse oximetry monitoring. To the best of our knowledge, this is the first study to include both a retrospective phase and a prospective validation phase of such a technique, in a wide number of patients and a real-life clinical situation.

Due to the ageing population, respiratory failure is a major public health concern and is burdened with a poor prognosis and a source of prolonged hospitalizations [28]. Early detection and prediction of organ failure (i.e. respiratory failure) could reduce health costs and risks for the patient, offering a reaction early and appropriate medical technology. RR is among the first vital signs to change in deteriorating patients. Adult general ward patients with a RR greater than 24 c/in should be monitored closely and reviewed regularly, even if the other vital signs are normal [29]. A patient with a RR greater than 27 b/min should receive immediate medical review. Intermittent RR measurement via visual observation is routine in many patient care settings; this approach is limited because it is intermittent (could lead to false appreciation of the real patient’s condition by spot check measurements), susceptible to significant human error [30], and requires clinical resources. Transthoracic impedance using ECG electrodes is a popular technique for continuous RR monitoring. This method obtains the respiration signals from the ECG electrodes rather than from the ECG signal. A limitation of thoracic impedance is that a movement artefact introduces errors in RR estimation. It is also sensitive to electrode misplacement and requires constant ECG monitoring which is not ubiquitous in general wards. While used in routine in the operating and recovery room, capnography has also well-documented technical and physiological limitation for spontaneously breathing patients [21], with a high incidence of signal unavailability. However, RR monitoring usually requires biomedical monitoring devices that are not available in clinical routine (ECG continuous monitoring, capnography, acoustimetry, etc.) or human intervention for chronometric measurements. Other continuous monitoring techniques are of limited interest in standard clinical settings either because of the cost and/or availability of the monitoring devices (ex. ECG monitoring or acoustimetry) or because of some technical flaws limiting the availability of the monitoring [31]. The integration of a RR estimation method inside a biomedical technique that is universally used in clinical routine may therefore enable safety improvement. The development of automated RR monitoring modalities may provide more accurate and objective measurements of this vital sign [32]. In such condition, PPG is an interesting approach for ventilation monitoring, as this technique would make simultaneous monitoring of RR and arterial oxygen saturation (pulse oximetry) possible, thus minimizing the number of sensors attached to the patient.

In a study involving 79 patients and volunteers (53 patients and 26 volunteers) aiming to evaluate such a software approach, the authors demonstrated a good correlation between PP RR evaluation and the reference value, with a low bias and limit of agreement [21]. Despite promising results, main limitation of this study was the low number of included patients, the fact that they did not seem to experience acute respiratory failure (mean RR = 16.0 ± 4.6 b/min), and a peak-by-peak comparison, thus limiting discrepancy related to artefacts on continuous measurements. 12.3% of data were excluded from analysis with respect to such interferences, while our results are provided on the entire group of patients and during complete analysis periods, without any data exclusion. The PPG is considered to be very sensitive to any irregularity of the pulse [33]. It means that PPG-RR monitoring may not be available and/or less reliable under certain conditions, such as heart arrhythmia, pacemaker, weak PPG signal and/or excessive movement artefacts.

Considering artefact detection and treatment that were introduced in our algorithm, PPG-RR monitoring remained reliable in 32 patients with atrial fibrillation (20.6 ± 3.7 and 20.5 ± 4.0; *R* = 0.916) during the retrospective evaluation phase, even if values were not available for some patients. Correlation with reference was high (*r* = 0.95, *p* < 0.0001), with a low bias (0.1 b/min) and limit of agreement (96% SD; − 3.3 to + 3.5 b/min), similar to various techniques [21, 31]. However, while remaining within a range of good acceptability (ICC = 0.689) [34], intraclass correlation was lower in case of AF and some patients experienced higher estimation errors. Such results warrant caution before generalization of the method in patients with atrial fibrillation.

Unlike previous studies that estimated respiratory rate in experimental settings or modified the clinical workflow, in the absence of activity or critical condition, the main strength of this study is that it was undertaken in a clinical setting with no modification to their existing clinical workflow and without the introduction of any additional sensor to be attached to the patient, *a contrario* to either capnography or acoustic methods. The other strength of the study is that during both the retrospective data warehousing and the prospective evaluation phases, a huge number of patients were recorded independently of their clinical status and the existence of artefacts, while in various other methods, evaluations of mostly healthy volunteers and/or a small number of patients were recorded. The evaluation method also seems to be independent of respiratory drive, while values were reliable for spontaneously and ventilated patients, as for patients with low or high tidal volume. We thus do consider that the current study conditions are much close to its real bedside application.

The first limit of the study that may be considered is the fact that *a contrario* to other validation studies for different RR measurement devices and/or algorithms, we chose to compare mean RR values over a clinically relevant period, instead of comparing peak-by-peak values. Such a method was chosen considering that what is really important is the mean RR trend over a significant time period, instead of point-check evaluations; doing this, we also emphasized the potential flaws of our algorithm and increased estimated RR standard deviation, while including periods of time where several artefacts and/or patients’ movements may have increase difficulties to get valid data. We therefore consider that the final device and algorithm were tested in a more clinically relevant situation, instead of doing experimental comparisons solely. Within our algorithm, RR estimation quality was assessed using the standard SpO_2_ signal quality index. Further improvement in the estimation accuracy will probably require dedicated artefact detection indexes. A second limit of the proposed method is that, even if quite independent from respiratory drive, PPG-RR monitoring is only reliable when enough evidence on the respiratory component is depicted on the PPG signal during the analysis windows. However, Simoes et al. [35] compared the respiratory rate estimated by manual counting with that obtained using electrical impedance, with chest electrodes. They found that the standard deviation of the difference between respiratory rates estimated using manual counting and that obtained using chest electrodes was 8.6 breaths per minute, with a mean difference of 1.72 breaths per minute. As mentioned earlier, after repeating this calculation for our data, we obtained the same value for standard deviation with a slightly lower mean value. This suggests that the variance of the difference between respiratory rate estimated by manual counting and using electrical impedance measurements using chest electrodes is comparable to the difference between respiratory rates obtained by manual counting and that obtained with our algorithm for processing the PPG waveform. Moreover, even if the limit of agreement of our method was higher in the prospective evaluation phase on 30 patients (96% SD; − 4.5 and + 5.5 c/min), with a low bias (0.5 c/min), it is well below to what is obtained with electrical impedance [31]. Last limitation of the PPG-RR estimation is the fact that it requires a certain level of curves visualization. Exclusion of patients with peripheral vasoconstriction (e.g. hemodynamic instability an unavailability of the PPG curve) may have induced a selection bias and a potential overestimation of the device’s performance.

## Conclusion

Our work presents an algorithm for respiratory rate estimation, which uses PPG. PPG is an interesting approach for ventilation and oxygenation monitoring, as this technique would make simultaneous monitoring of respiratory rate and oxygen saturation (pulse oximetry) possible, minimizing the number of sensors attached to the patient in general wards. The algorithm was validated on a great number of patients with various pathologies and a wide range of respiratory rates. It can be used as a reliable adjunct to pulse oximetry monitoring, in order to assess patients’ clinical status.

## Figures and Tables

**Fig. 3:**
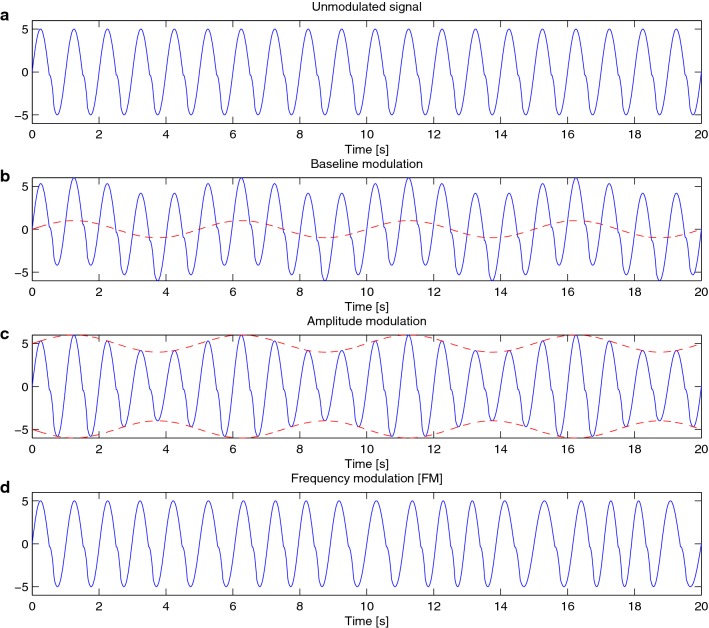
Three principal types of respiratory modulation of the plethysmogram signal (PPG) that are used for respiratory rate estimation. **a** Unmodulated PPG waveform. **b** Baseline modulation [BM]; the red dashed line depicts the baseline modulation related to the respiratory rate component. **c** Amplitude modulation (AM); the two red dashed lines depict the signal envelope related to the respiratory rate component. **d** Frequency modulation (FM); this modulation of the heart rate, also called “respiratory sinus arrythmia”, is synchronized with the respiratory rate

## Appendix

See Fig. 3.

## Abbreviations

AM: amplitude modulation
FM: frequency modulation
ICU: intensive care unit
RR: respiratory rate
ECG-RR: respiratory rate derived from electric thoracic impedance
PPG-RR: respiratory rate derived from the photoplethysmographic signal
Manual-RR: respiratory rate measured by chronometric evaluation
SAPS II: version 2 of the Simplified Acute Physiology Score
